# AIE-active non-conjugated poly(*N*-vinylcaprolactam) as a fluorescent thermometer for intracellular temperature imaging†
†Electronic supplementary information (ESI) available. See DOI: 10.1039/c9sc04338a


**DOI:** 10.1039/c9sc04338a

**Published:** 2019-10-28

**Authors:** Biswajit Saha, Bhuban Ruidas, Sourav Mete, Chitrangada Das Mukhopadhyay, Kamal Bauri, Priyadarsi De

**Affiliations:** a Polymer Research Centre and Centre for Advanced Functional Materials , Department of Chemical Sciences , Indian Institute of Science Education and Research Kolkata , Nadia , Mohanpur – 741246 , West Bengal , India . Email: p_de@iiserkol.ac.in; b Centre for Healthcare Science and Technology , Indian Institute of Engineering Science and Technology , Shibpur, P.O. Botanic Garden , Howrah , West Bengal 711103 , India . Email: chitrangadam@chest.iiests.ac.in; c Department of Chemistry , Raghunathpur College , Purulia – 723133 , West Bengal , India . Email: kamalsom98@gmail.com

## Abstract

Since temperature is one of the most significant physiological parameters that dictate the cellular status of living organisms, accurate intracellular temperature measurement is crucial and a valuable biomarker for the diagnosis and treatment of diseases. Herein, we introduce the foremost example of a non-conjugated polymer as a next generation fluorescent thermometer which is capable of addressing the key shortcomings including toxicity and thermal-induced fluorescence quenching associated with π–π conjugated system-based thermometers developed so far. We revealed, for the first time, the unique photophysical and aggregation-induced emission (AIE) characteristics of well-known thermoresponsive poly(*N*-vinylcaprolactam) (PNVCL) devoid of any classical fluorophore entity. PNVCL underwent a coil to globular conformational transition in an aqueous medium and appeared to be fluorescent above its lower critical solution temperature (LCST) near body temperature (38 °C). Eventually, this intriguing aspect enabled higher cellular uptake of PNVCL at the LCST boundary. By virtue of the AIE effect, the thermo-induced aggregation phenomenon has been ingeniously utilized to apply PNVCL as a novel fluorescent thermometer for intracellular temperature determination.

## Abstract 2

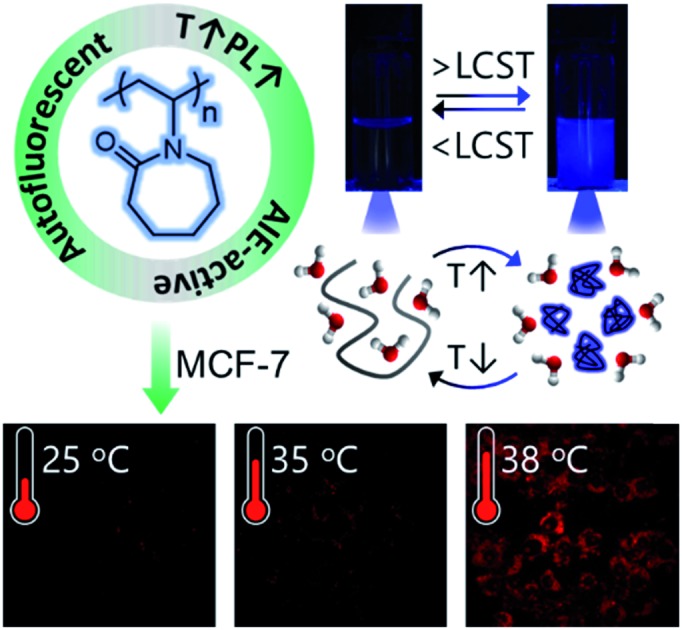
Unprecedented photoluminescence, AIE and heat-induced emission characteristics of non-conjugated thermoresponsive PNVCL were unveiled for the very first time which eventually empowered PNVCL to act as an intracellular thermometer.

## Introduction

Temperature is an important physiological parameter that governs a broad range of biological activities, especially all biological reactions within living cells.^1^,^2^ In addition, various kinds of cellular disorders, such as inflammation and cancer cell growth, are often accompanied by a temperature increase, an indication of microenvironment dysfunction.^3^–^6^ Therefore, the development of a thermo-sensitive device or a thermometer with the ability to detect and monitor the intracellular temperature with high sensitivity is highly desired. Obviously, accurate intracellular temperature mapping of living cells should represent a novel tool for analysis of the cellular status and contribute to the establishment of novel diagnostics and therapies.

In this context, an astonishing evolution of numerous fluorescent thermometers has been witnessed over the past few years.^7^,^8^ However, such molecular fluorescent thermometers often exhibit many limitations including poor hydrophilicity, undesired leakage from the cell, weak structural stability, and non-negligible biotoxicity. To overcome these shortcomings, a number of fluorescent polymeric thermometers have been designed and applied to monitor the temperature inside living cells due to their improved photostability, water stability and biocompatibility.^9^,^10^ Generally, fluorescent polymeric thermometers comprise a thermo-responsive unit and a polarity-sensitive conventional fluorophore unit, which could typically be incorporated either through copolymerization of a fluorescent-tagged monomer or by post-polymerization modification. The variation of the fluorescence intensity or lifetime of such polymers is mostly associated with the temperature-driven conformational transformation of the thermo-sensitive unit that alters the microenvironmental polarity of the fluorescent moieties embedded within the polymer chain. However, bright emission is always observed from a conventional conjugated fluorophore attached to a polymer, but it is usually weakened or quenched in the concentrated or in the aggregated state, frequently referred to as “aggregation-caused quenching” (ACQ).^11^

The discovery of the aggregation-induced emission (AIE) phenomenon by Tang's group^12^ has tackled the problematic aggregation-caused quenching (ACQ) effect of conventional chromophores and has shown significant academic value and promising applications in cell imaging, fluorescent sensors, and bio-probe materials.^13^,^14^ Until now, although multitudinous AIE-active molecules have been designed and synthesized, very few have been integrated with the thermo-responsive system to construct a fluorescent thermometer with AIE characteristics for detection and monitoring of the variation of intracellular temperature at the microscale.^15^,^16^ Despite having reasonable significance, the aforementioned polymeric fluorescent thermometers have some inherent disadvantages: (i) a tedious multi-step synthetic procedure is needed to synthesize such systems; (ii) these AIE-active moieties with π–π conjugated structures might lead to a lot of restrictions for biological applications namely high cytotoxicity, acute inflammation and serious immunogenicity.^17^ These complications have motivated us to develop an alternative design that might overcome these bottlenecks of conjugated compounds. In this regard, the design of a π–π conjugation-free polymer based fluorescent thermometer containing non-conventional fluorophores with thermo-induced AIE characteristics which could eliminate the tedious synthetic steps has become imperative but has remained virtually unexplored. On the other hand, Uchiyama and co-workers have fabricated an elegant fluorescence lifetime-based thermometer for mapping intracellular temperature changes at the subcellular scale using fluorescence lifetime imaging microscopy.^18^ Fluorescence lifetime imaging requires a relatively longer time (1 min) to collect one image, whereas a fluorescence intensity image could be collected within 1 second.^19^ Hence, it should be noted that fluorescence intensity-based thermometers are much easier to access compared to fluorescence lifetime-based thermometers.^20^

Electron-rich heteroatoms, such as nitrogen, oxygen, phosphorous, sulfur, and/or unsaturated C=C, C=O and C≡N functionalities, have been reported to act as nonconventional luminophores upon aggregation or clustering.^21^–^23^ We hypothesized that by virtue of structural flexibility, temperature sensitivity and electron-rich heteroatoms (N and O), non-conjugated poly(*N*-vinylcaprolactam) (PNVCL) is expected to exhibit interesting photophysical behavior. As anticipated, for the very first time, we have revealed the intrinsic photoluminescence characteristics of well-known thermo-responsive PNVCL and its AIE activity. The temperature-driven aggregation behavior and AIE characteristics have judiciously been exploited to use PNVCL as a potential fluorescent polymeric thermometer for intracellular temperature measurement. Overall, the present non-conjugated macromolecule has the ability to replace the current traditional-fluorophore functionalized fluorescent polymeric thermometer, because PNVCL has multiple fascinating features such as AIE, temperature-sensitive fluorescence enhancement, and excellent cytocompatibility, which are rare in a single polymeric system.

## Results and discussion

**Scheme 1:**
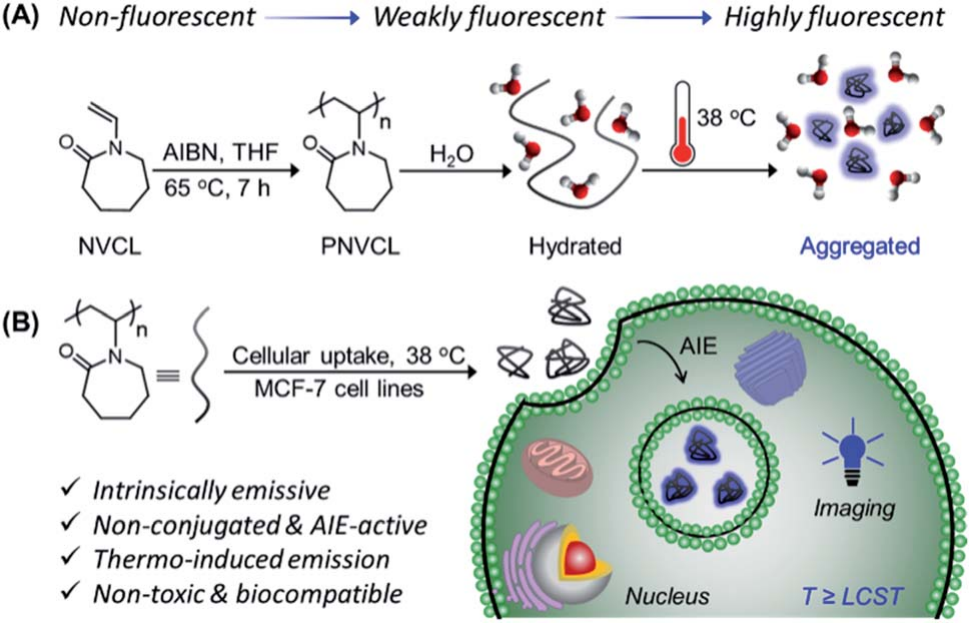
(A) Synthesis of PNVCL *via* FRP and its thermo-induced conformational transformation. (B) Overall schematic illustration of intracellular temperature imaging using non-conjugated PNVCL as a fluorescent thermometer.

Initially, PNVCL was synthesized in one step by conventional free radical polymerization (FRP) of commercially available *N*-vinylcaprolactam (Scheme 1A). ^1^H NMR spectroscopy and size exclusion chromatography (SEC) were employed to characterize PNVCL. A typical ^1^H NMR spectrum shown in Fig. S1† clearly shows all the characteristic signals corresponding to the polymeric structure. The purified polymer exhibited a number average molecular weight (*M*_n,SEC_) of 9500 g mol^–1^, with dispersity (*Ð*) = 1.95 (Fig. S2†). PNVCL was found to be soluble in various organic solvents such as tetrahydrofuran (THF), *N*,*N*-dimethylformamide (DMF), dioxane, methanol (MeOH), ethanol (EtOH), acetone, *etc.*, except in hexane. Moreover, the polymer was soluble in aqueous media owing to the strong H-bonding between the C=O groups of lactam and water molecules.

**Fig. 1:**
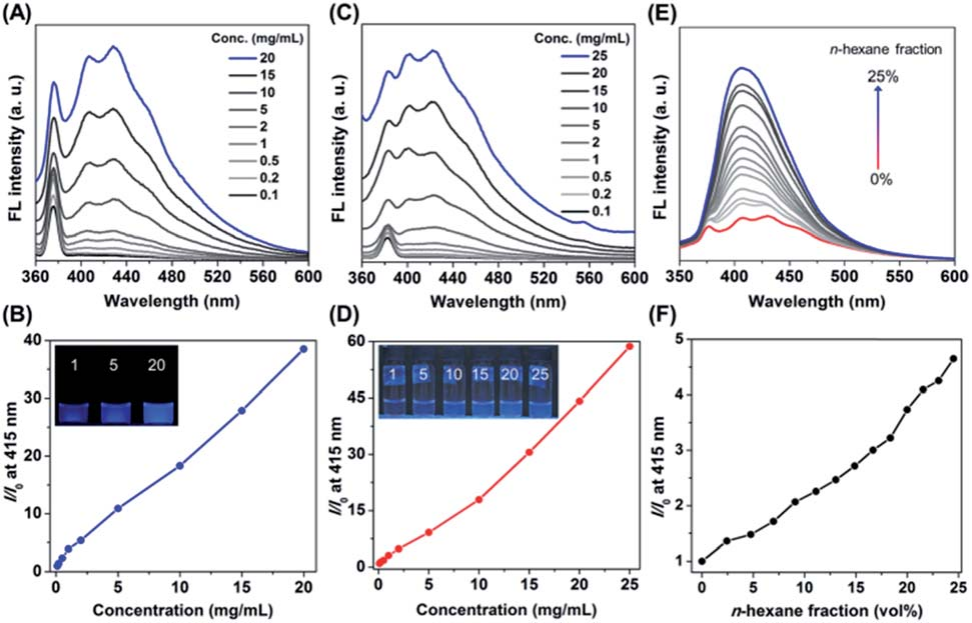
AIE features of PNVCL. (A) Concentration-dependent PL spectra and (B) the corresponding plot of intensity (at 415 nm) ratios (*I*/*I*_0_) *versus* polymer concentration in THF. Inset: digital images of 1, 5, and 20 mg mL^–1^ PNVCL in THF obtained under 365 nm UV illumination. (C) Concentration-dependent PL spectra and (D) the plot of *I*/*I*_0_*versus* PNVCL concentration in water. The inset shows images of PNVCL solutions of different concentrations (mg mL^–1^) upon illumination with a 365 nm UV lamp. (E) PL spectra of PNVCL in THF/*n*-hexane mixtures with increasing *n*-hexane fractions at room temperature. (F) Relationship between relative PL intensity *I*/*I*_0_ and *n*-hexane fractions present in THF/*n*-hexane mixtures, where *I*_0_ is the PL intensity in pure THF and *I* is the change in intensity for various *n*-hexane fractions.

In order to explore the photophysical behaviour of PNVCL, UV-Vis absorption spectra of the polymer were recorded in different solvents (Fig. S3†). PNVCL showed an absorption maximum at 236 nm in THF, assigned to the π–π* transition of the carbonyl group. Next, the polymer solutions were excited with an excitation wavelength (*λ*_ex_) of 339 nm in different solvents to understand their emission characteristics. As illustrated in Fig. S4,† PNVCL (2 mg mL^–1^) exhibited stronger fluorescence in THF than in any other common organic/aqueous solvents. Thus, PNVCL possessed a higher quantum efficiency (*φ*_F_ at *λ*_ex_ 350 nm) and average fluorescence lifetime ( 350 nm) and average fluorescence lifetime (〈*τ*〉) in THF () in THF (*φ*_F_ = 4.1%, *τ* = 6.17 ns) than in water (*φ*_F_ = 0.2%, = 0.2%, 〈*τ*〉 = 5.86 ns) (Fig. S5 = 5.86 ns) (Fig. S5†). A dilute PNVCL solution (0.1 mg mL^–1^ in THF) yielded a low photoluminescence (PL) signal with an almost flat line parallel to the abscissa and obviously no emission was observed upon irradiation (Fig. 1A). But with an increasing polymer concentration, the PL intensity was found to increase gradually. About a 39-fold increase of the emission intensity at *λ*_em_ = 415 nm was observed when the concentration was increased from 0.1 to 20 mg mL^–1^ (Fig. 1B). A similar tendency was observed in water as well (Fig. 1C), with a much higher PL intensity enhancement (∼59 fold) in comparison to that with the THF solution (Fig. 1D). As the solution became more concentrated, it emitted much brighter blue light in both THF and water upon UV illumination (inset of Fig. 1B and D). This concentration-dependent enhanced emission of PNVCL is exactly opposite to the concentration-dependent quenching phenomenon usually observed in traditional luminogens. The AIE behavior of the polymer was further assessed in a THF/*n*-hexane mixture at a concentration of 20 mg mL^–1^. Interestingly, enhancement of the PL intensity was observed upon incremental addition of the bad solvent (*n*-hexane) to the good solvent (THF) containing the polymer, without increasing the polymer concentration (Fig. 1E). The highest emission intensity was observed in 25 vol% *n*-hexane with a 5-fold enhancement as compared to that of pure THF solutions (Fig. 1F).

Although the present polymer lacks conventional chromophores, *i.e.* any sort of π-conjugated system, it produces bright blue emission in the concentrated or aggregated state. This fact can be attributed to the clustering-triggered emission (CTE) mechanism.^24^ This “clusteroluminescence” coined by Tang's group is a common phenomenon in the case of nonconventional luminogens containing heteroatoms with a lone pair of electrons or specific functionality such as C=C, C=O, C≡N, *etc.*^24^ In this scenario, for PNVCL, the nonconventional chromophore moiety namely C=O groups with π-electrons/a lone pair of electrons and N atoms with a lone pair of electrons of one cyclic amide come in close proximity to another amide to form a cluster in the aggregated state. This clustering results in chromophores with effective through-space electronic communication giving rise to extended electron delocalization and simultaneous rigidified conformation. The rigidity of the molecular conformation restricts vibration and molecular rotation which efficiently suppresses the non-radiative relaxation.^25^ Consequently, the polymer exhibits noticeable emission upon irradiation in the aggregated state.

**Fig. 2:**
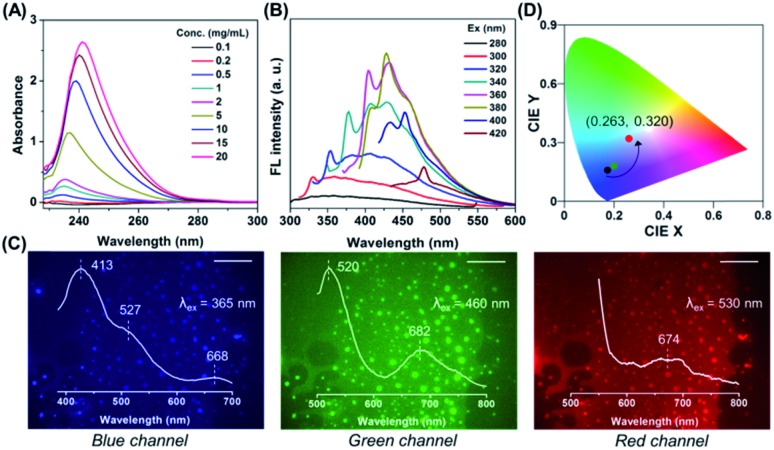
(A) Absorption spectra of PNVCL at different concentrations in THF. (B) PL spectra of PNVCL recorded at different excitation wavelengths λ_ex_. Concentration = 20 mg mL^–1^ in THF. (C) Fluorescence microscopy images of the PNVCL film obtained from the blue (420–460 nm), green (510–550 nm) and red (575–625 nm) emission channels, respectively. Emission spectra of the PNVCL film with different λ_ex_ values are also included in each photograph. Scale bars = 50 μm. (D) The emission regions of fluorescent PNVCL in THF (black dot) and water (green dot) and in the solid state (red dot) assigned in the CIE_*x*,*y*_ chromaticity diagram.

Notably, progressive enhanced absorption with red shifting of absorption maxima was observed as the polymer concentration increased (Fig. 2A), indicating the formation of certain aggregates. Furthermore, with the increasing excitation wavelength, the emission maximum was found to be red-shifted accompanied by emission intensity enhancement (Fig. 2B and S6†). This concentration-dependent absorption profile and excitation-dependent fluorescence (EDF) phenomenon certainly indicated the existence of various stable excited states with many different energy levels in the aggregated state. Again this EDF phenomenon is common for non-traditional luminogens with clustering triggered emission characteristics^26^ and distinguishable from the classic fluorophore system with relatively fixed emission as the intrinsic characteristic.^27^ Indeed, this suggests that the fluorescence of a non-conjugated system in the aggregated state originated not from a specific entity with a definite structure but plausibly from interaction among chromophores with heterogeneity and complexity.^28^,^29^

To further shed light on this CTE process, we examined the emission behavior of a PNVCL film (solid state), where there is more opportunity for the chromophores to approach closer and provide discernible emission. Surprisingly, direct observation of the PNVCL film under a fluorescence microscope showed tunable photoluminescence from blue and green to red upon altering the emission channels (Fig. 2C). Furthermore, the variation of the emission profile and emission maxima in the PL spectra recorded with *λ*_ex_ values of 365 (UV), 460 (blue) and 530 nm (green) was consistent with the preceding observations (inset of Fig. 2C). Apart from this, in the solid state PNVCL emitted close to the white-light equi-energy point (CIE coordinates (0.263, 0.320), red dot), while in the solution state it emitted pure blue light (Fig. 2D, black and green dots). This result further coincides with the observation made using fluorescence microscopy images. Thus, the EDF phenomenon associated with the CTE process unequivocally confirmed the presence of various stable excited states with many different energy levels.

**Fig. 3:**
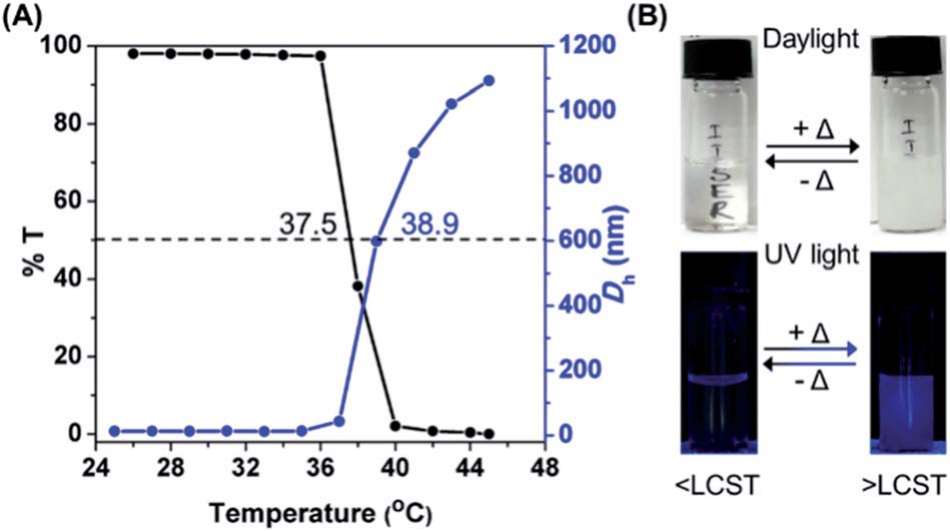
(A) Variation of %*T* and *D*_h_ of a 0.1 wt% aqueous PNVCL solution as a function of temperature. (B) Photographs of the same PNVCL solution acquired under daylight and UV irradiation (365 nm) below and above the LCST.

PNVCL is a well-reported water-soluble polymer with an appealing temperature-dependent phase transition behavior (Scheme 1A), which generally plays a crucial role in fluorescence activity.^30^,^31^ The thermal response of PNVCL was investigated based on changes in its solution transmittance at 500 nm *via* UV-Vis spectroscopy.^32^ No discernible change in transmittance (%*T*) was observed until 35 °C; an abrupt decrease due to the LCST was observed at 37.5 °C (Fig. 3A). This is because of the rupturing of hydrogen bonding between the C=O groups of lactam units and O–H groups of water molecules at a higher temperature.^33^ Moreover, variations in the average hydrodynamic diameters (*D*_h_) of the polymer in response to heat were assessed by DLS measurements below and above the LCST. The *D*_h_ values in the temperature window 25–35 °C (below the LCST) were found to be 6–8 nm and increased up to 170–1000 nm above the LCST (Fig. 3A), signifying the transformation of the polymer conformation from a random coil to large globular microparticles (Fig. S7†). The synthesized PNVCL showed an LCST near body temperature, *i.e.* 37.5 °C; thus it is an appropriate candidate for biological applications.^34^ Subsequently, the effect of temperature on the optical properties of PNVCL was investigated. As shown in Fig. S8,† the emission intensity increased upon heating and a reasonable enhancement in PL intensity was noticed at and above the LCST (≥38 °C).

Thus, we recognized that below the LCST, there was no observable luminescence in water at low polymer concentrations. Quite interestingly, at temperatures ≥ LCST an intense bright blue fluorescence was observed under UV light illumination of 365 nm (Fig. 3B). This was ascribed to the heat-induced conformational change from the coiled to the globular state, leading to AIE. This phenomenon was found to be reversible with respect to temperature (data not shown here).

Before application of PNVCL in a biological platform, we studied the cytotoxicity of the polymer against both breast cancer cells (MCF-7) and normal lung epithelial cells (WI-38) by performing MTT assay in a dose-dependent manner (Fig. S9†). The cell viability of PNVCL was above 80% in both MCF-7 and WI-38 cell lines at a concentration of 500 μg mL^–1^, implying its high biocompatibility.^35^

**Fig. 4:**
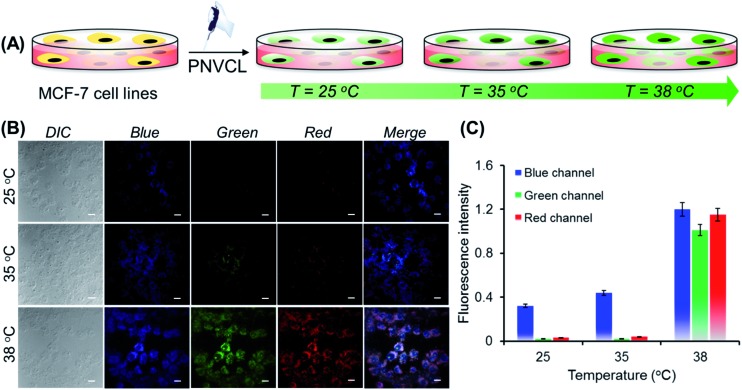
(A) Cartoon representation of MCF-7 cells co-cultured/incubated with autofluorescent PNVCL at different temperatures. (B) CLSM images of MCF-7 cells labelled with PNVCL (250 μg mL^–1^) at 25, 35, and 38 °C for 24 h. Scale bars = 10 μm. (C) Temperature governed fluorescence intensities of PNVCL treated MCF-7 cells were measured and expressed as a ratio of intensity of blue, green and red fluorescence in respect of blue fluorescence emitted by 4′,6-diamidino-2-phenylindole (DAPI) only. Data are expressed as the mean of triplicate determinations (mean ± standard error of mean (SEM)), represented by vertical bars (*P < 0.05).

Non-toxicity at a high dose, temperature-induced aggregation and AIE features of PNVCL have been utilized to investigate the potentiality of PNVCL to act as a polymeric fluorescent intracellular thermometer. The intracellular temperature measurement in MCF-7 cells using PNVCL was performed through confocal laser scanning microscopy (CLSM). Cultured cells after treatment with PNVCL were incubated for 24 h at 25, 35, and 38 °C (Fig. 4A). The CLSM images barely showed any green or red fluorescence inside the cells incubated at 25 and 35 °C (below the LCST), although weak blue fluorescence was visible at these temperatures. It is noteworthy that there was a remarkable light-up fluorescence signal inside the cells incubated above the LCST, *i.e.* 38 °C (Fig. 4B), when observed in all three channels (blue, green and red). This is in good agreement with the previous temperature-induced fluorescence enhancement phenomenon (Fig. 3B). However, a slight increase in fluorescence intensity was observed in all three emission channels beyond 38 °C (Fig. S10†).

As shown in Fig. 4C, approximately 50 and 29-fold enhancements of green and red fluorescence intensity were detected, respectively, when the temperature was altered by 3 degrees in the vicinity of the LCST from 35 to 38 °C. The above outcomes implied that the cellular internalization of the non-conjugated PNVCL fluorescent probe is indeed controlled by the temperature of the culture medium (Scheme 1B). The higher cellular internalization of the present polymer above the LCST seems to be because of two plausible mechanisms. Firstly, there will be increasing hydrophobic interaction between the cell membranes and the hydrophobic polymer chains, caused by the dehydration of the hydrated polymer chain above the LCST, while there will be decreasing cell membrane and hydrated polymer chain interaction below the LCST.^36^ Consequently, the cellular uptake of the polymer was improved above the LCST. Secondly, above the LCST extended coiled polymer chains collapsed into globular aggregates, which spontaneously internalized through improved interaction with the cell membrane.^37^ These results unambiguously demonstrate the capability of PNVCL as a fluorescent thermometer for detecting the intracellular temperature change. It is noteworthy that no changes in the fluorescence response of PNVCL were observed upon varying the environmental pH from 4 to 10 (Fig. S11†), certifying its high thermal-sensing accuracy.

**Fig. 5:**
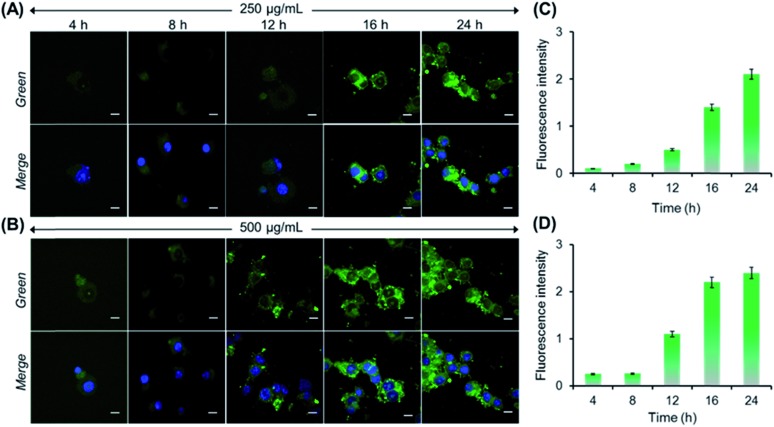
CLSM images of MCF-7 cells after incubating with 250 μg mL^–1^ (A) and 500 μg mL^–1^ (B) PNVCL for different time intervals at 38 °C (scale bar is 10 μm). Effect of incubation time on the fluorescence intensity of MCF-7 cells stained with PNVCL at two different concentrations, 250 μg mL^–1^ (C) and 500 μg mL^–1^ (D). Intensities were quantitatively expressed as a ratio of intensity of green fluorescence relative to that of blue fluorescence emitted by DAPI only.

Next, we performed both concentration and incubation time-dependent cellular uptake studies to demonstrate the AIE activity of PNVCL inside the cells. Initially, MCF-7 cells were treated with PNVCL at a concentration of either 250 or 500 μg mL^–1^ for different incubation times followed by staining of nuclei using DAPI (blue emission). At 38 °C (above the LCST) and at a fixed polymer concentration (250 μg mL^–1^), with an increasing incubation time ranging from 4 to 24 h the average emission intensity obtained from MCF-7 cells observed under the green channel was found to increase (Fig. 5A). This indicated a gradual increase of polymer uptake by the cells upon prolonging the incubation time. A similar tendency was observed when these cells were treated with 500 μg mL^–1^ polymer containing medium (Fig. 5B). CLSM images clearly showed that the polymer localized mainly in the cytoplasm of the cells without transferring to the nucleus, as no colocalization of the fluorescence signal coming from PNVCL with that from DAPI (a commercial cell nucleus tracker) was found in overlay images. Again for the same incubation time, the cells treated with a higher concentration of the polymer containing medium showed more intense green fluorescence than the cells treated with lower polymer concentrations (Fig. 5C and D).^38^ Altogether, the above findings clearly demonstrated the AIE-activity of the present polymer inside the cells, as fluorescence intensity was generally suppressed or quenched in cells stained with classic fluorophores due to ACQ upon prolonging the incubation time.^39^

## Conclusions

In summary, unique emission behaviour of non-conjugated PNVCL without traditional chromophores has been investigated. Although PNVCL is non-fluorescent in dilute solution (up to 0.1 mg mL^–1^), it exhibited significant emission upon increasing the concentration, and also in the solid and film state. This unprecedented light emission could be attributed to the AIE or CTE mechanism. Interestingly, the PNVCL film displayed distinct blue, green and red emission upon illumination with different excitation wavelengths, demonstrating the coexistence of multiple emissive species with different energy levels. Moreover, the PNVCL solution exhibited a bright blue emission upon heating above its LCST (37.5 °C), which was attributed to the heat-assisted aggregation-induced emission phenomenon. Capitalizing on the aforesaid aspects, a higher cellular internalization of PNVCL was attained at 38 °C (above the LCST) compared to 35 °C (below the LCST) with significant light-up fluorescence confirmed through CLSM images. We believe that the present findings disclosed the potentiality of the non-toxic biocompatible PNVCL to be a next generation fluorescent thermometer by detecting minor temperature changes, beneficial for the early detection of diseases and local hyperthermia treatment.^40^

## Conflicts of interest

There are no conflicts to declare.

## Supplementary Material

Supplementary information
