# Supplementary material for: AIE-active non-conjugated poly(N-vinylcaprolactam) as a fluorescent thermometer for intracellular temperature imaging
Source: Chem Sci. 2019 Oct 28;11(1):141–7. doi: 10.1039/c9sc04338a (PMC7012023; doi:10.1039/c9sc04338a)
Supplement: Supplementary file 1 [file SC-011-C9SC04338A-s001.pdf]

Electronic Supplementary Material (ESI) for Chemical Science.
This journal is © The Royal Society of Chemistry 2019

Supporting Information for

AIE-active non-conjugated poly(*N*-vinylcaprolactam) as fluorescent thermometer for intracellular temperature imaging

Biswajit Saha,[a] Bhuban Ruidas,[b] Sourav Mete,[a] Chitrangada Das Mukhopadhyay,*[,b] Kamal Bauri,*[,c] and Priyadarsi De*[,a]

[a]Polymer Research Centre and Centre for Advanced Functional Materials, Department of Chemical Sciences, Indian Institute of Science Education and Research Kolkata, Mohanpur - 741246, Nadia, West Bengal, India

[b]Centre for Healthcare Science and Technology, Indian Institute of Engineering Science and Technology, Shibpur, P.O. Botanic Garden, Howrah, West Bengal, 711103, India

[c]Department of Chemistry, Raghunathpur College, Purulia - 723133, West Bengal, India

* Corresponding Authors: chitrangadam@chest.iiests.ac.in (CDM); kamalsom98@gmail.com (KB) ; p_de@iiserkol.ac.in (PD)

Experimental Details

Materials. *N*-vinylcaprolactam (NVCL) was purchased from Sigma-Aldrich and purified by recrystallization from methanol thrice and dried under vacuum at room temperature. HPLC water (Sisco Research Laboratories Pvt. Ltd., India), 4',6-diamidino-2-phenylindole (DAPI, Thermo Fisher Scientific), 3-(4,5-dimethylthiazol-2-yl)-2,5-diphenyltetrazolium bromide (MTT, USB Corporation) and 2,2'-azobis-(2-methylpropionitrile) (AIBN, Sigma) were used as received, except AIBN, which was utilized as an initiator after recrystallization in methanol. Anhydrous tetrahydrofuran (THF) was

prepared by following standard literature protocol.[1] NMR solvent, $CDCl_3$ was bought from Cambridge Isotope Laboratories, Inc., USA. *N,N'*-dimethylformamide (DMF), dimethyl sulfoxide (DMSO), methanol (MeOH), 1,4-dioxane were obtained from Merck and used without any further purification.

Instruments and Characterizations

Proton Nuclear magnetic resonance (^{1}H NMR). ^{1}H NMR spectrum of the polymer was recorded with a Bruker AvanceIII spectrometer operating at 500 MHz in $CDCl_3$ at 25 ^{o}C.

Size exclusion chromatography (SEC). SEC was employed to obtain the molecular weight and dispersity ($Đ$) of polymer in DMF solvent with 0.8 mL/min flow rate at 40 ^{o}C. The instrument contains a Waters 1515 HPLC pump, Waters 2414 refractive index (RI) detector, one styragel HR 4E column (7.8 × 300 mm) and one styragel HR3 column (7.8 × 300 mm). Poly(methyl methacrylate) (PMMA) standards were used to calibrate the instrument.

UV-Vis spectroscopy. Absorption spectra of polymer solutions were recorded between 200 and 500 nm wavelength with a Perkin-Elmer Lambda 35 spectrophotometer. Temperature-dependent optical properties of aqueous polymer solutions were measured with the help of a Peltier temperature controller connected with the spectrophotometer.

Fluorescence spectroscopy. Fluorescence emission spectra were monitored using a Horiba JobinYvon (Fluoromax-3, Xe-150 W, 250−900 nm) fluorescence spectrometer. The fluorescence quantum yield (ϕ_F) in solution was determined using quinine sulfate in 0.05 M sulphuric acid (ϕ_F = 52%) as a standard.

Fluorescence lifetime measurements. Fluorescence lifetime measurements were conducted by the method of time-correlated single-photon counting (TCSPC) using a picoseconds spectrofluorimeter from Horiba JobinYvon IBH equipped with a FluoroHub

single photon counting controller, Fluoro3PS precision photomultiplier power supply, and FC-MCP-50SCMCP-PMT detection unit. The 340 nm laser head was applied as the excitation source.

Dynamic light scattering (DLS). Size distribution measurements were carried out in a Malvern Nano Zetasizer instrument equipped with a He-Ne laser (λ = 633 nm) and scattering angle of 173°. Polymer solutions were filtered through a 0.45 μm syringe filter prior to measurement.

Fluorescence microscope. An epifluorescence Olympus IX81 model equipped with plan fluorite objective with 60× magnification was used to obtain fluorescence images of PNVCL film.

Synthesis of poly(*N*-vinylcaprolactam). Briefly, NVCL (3.0 g, 21.5 mmol) and AIBN (3.53 mg, 21.5 μmol) were dissolved in dry THF (10 mL) and charged into a 20 mL septa-sealed reaction vial equipped with a magnetic stirring bar at a feed ratio of 50:0.5. The reaction vial was carefully purged with dry N_2 for 10 min at 0 °C and subsequently placed on a preheated reaction block at 65 °C. After 7 h, the polymerization reaction was quenched by transferring the vial to an ice-water bath followed by exposing to air. The reaction mixture was precipitated into an excess of hexanes and dissolved it with acetone again. This dissolution-precipitation process with hexanes/acetone mixture was repeated for 8 times to remove unreacted monomer and other impurities. Finally, the purified polymer PNVCL was isolated as a white powder after overnight drying under high vacuum at room temperature. We obtained 2.4 g PNVCL (gravimetric conversion = 80 %).

Determination of LCST of aqueous PNVCL solution. In a typical procedure, 0.1 wt % of a transparent aqueous solution of PNVCL was taken in 4.0 mL quartz cuvette and placed into a UV-Vis spectrophotometer equipped with a Peltier temperature controller. The turbidity of the solution was then measured by recording the percentage of transmittance

(%T) values at 500 nm in the temperature range of 20-42 °C with increasing 2 °C temperatures for each data after equilibrating for 5 min at the experimental temperature. LCST value was referred to the temperature at which %T was reduced to 50%.[2]

Furthermore, the LCST of PNVCL solution was determined by monitoring the abrupt change in the hydrodynamic diameters (D_hs) as a function of temperature using DLS instrument.

Cell culture. Human breast cancer cell line, MCF-7 and normal lung epithelial cell line, WI-38, were procured from the National Centre for Cell Science (NCCS), Pune, India and revived as recommended by the supplier. The above cell lines were cultured in Dulbecco Modified Eagles Media (DMEM) medium containing 10% fetal bovine serum (FBS), 100 μg/mL streptomycin, 100 I.U./mL penicillin, 100 μg/mL streptomycin and kept in an incubator at 37 °C in an atmosphere of 5% CO_2 and 95% air until they reached confluency. All the cell culture reagents were purchased from HiMedia, India.

Cytotoxicity evaluation of non-conjugated fluorescent PNVCL. The viability of MCF-7 and WI-38 cell lines after exposure to PNVCL at different concentrations (0-500 μg/mL)was determined by virtue of MTT (Invitrogen) assay.[3,4] In brief, cells were seeded in 96 well plates (1×10^5, 100 μL/well) in DMEM/FBS media and cultured for 24 h under 5% CO_2 at 37 °C. The media was then removed and fresh media supplemented along with a varied concentration of PNVCL and the cells were left undisturbed for next 24 h inside the CO_2 incubator. After incubation, 10 μL MTT reagent (5 mg/mL in PBS) was added in each well and allowed to incubate for 4 h. Next, 85 μL media was aspirated from each well and the resulting insoluble formazan crystals were dissolved in 50 μL DMSO. After 10 min, the absorbance of the formazan crystals was measured with the aid of a BioRad, iMark™ microplate reader at 570 nm wavelength. The media without cells was taken as a blank and

subtracted as background from each sample. The cell viability was calculated as $(A_{sample}/A_{control}) \times 100$; where A_{sample} and $A_{control}$ are the absorbance of sample well and control well, respectively.

Temperature and time-dependent cellular imaging. The MCF-7 cells were cultured on coverslips for one day at 37 ^{o}C in a humidified atmosphere containing 5% CO_2.The cultured cells were either mock-treated or treated with PNVCL (250 μg/mL) at 25, 35 and 38 ^{o}C for 24 h with 5% CO_2. Next, the treated cells were stained with DAPI (200 nM) and incubated for another 5-10 min at that respective temperature. Finally, the coverslips were washed with PBS buffer and mounted on a glass slide to observe under confocal laser scanning microscope (CLSM, Carl Zeiss) with 40X objective lens in blue, green and red channels, respectively.

A similar experiment was performed by varying incubation time (4-24 h) with two different polymer concentrations (250 and 500 μg/mL) at 38 ^{o}C. The DAPI staining was utilized to calculate the PNVCL emitted autofluorescence intensity.

Quantification of cellular fluorescence intensity. Quantitative measurement of cellular fluorescence intensity from microscopy images was done by using ImageJ software. Briefly, the cell of interest in a given microscopic field was outlined and measured for its intensity in the outlined area. This process was repeated for other cells in the field. Then a region neighbouring to the fluorescent cell but has no fluorescence was measured and set as background. Thereafter, all the data was pasted in a separate spreadsheet. Corrected total cell fluorescence (CTCF)can be obtained from the formula mentioned below.

CTCF = Integrated fluorescence density – (Area of selected fluorescent cell × Mean fluorescence of background readings)

In the present study, the fluorescence intensity of DAPI was considered to be independent of temperature and the intensity of PNVCL stained cells has temperature-dependent fluorescence. So the actual fluorescence was obtained quantitatively by normalizing with respect to the DAPI staining. Finally, the graphs were plotted to represent the change in intensity of PNVCL stained cells as a function of both temperature and time.

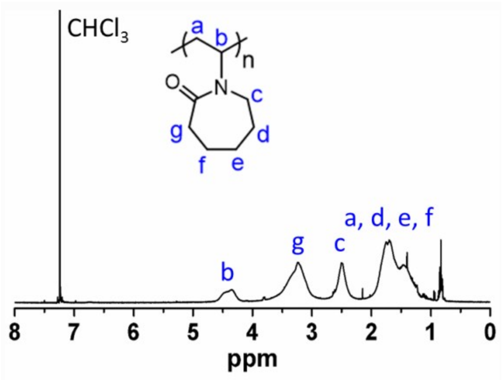

Fig. S1 ^{1}H NMR spectrum of PNVCL in $CDCl_3$.

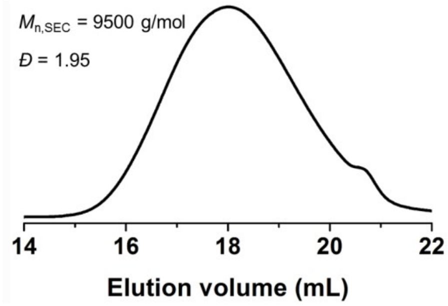

Fig. S2 SEC trace of PNVCL in DMF.

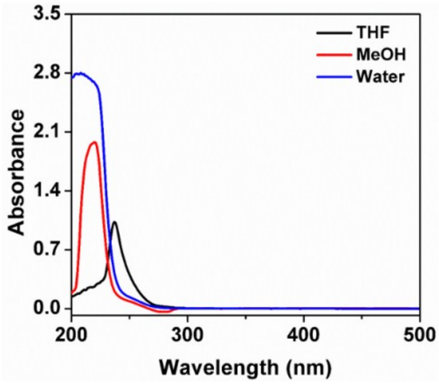

Fig. S3 UV-Vis absorption spectra of PNVCL in different solvents.

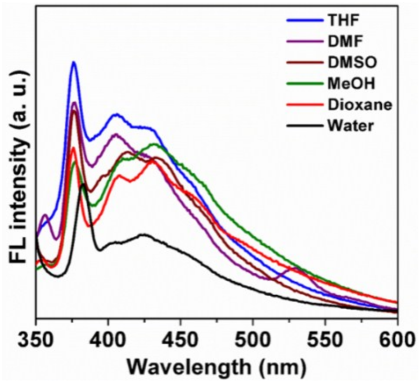

Fig. S4 Emission spectra of PNVCL in different solvents. λ_{ex} = 339 nm.

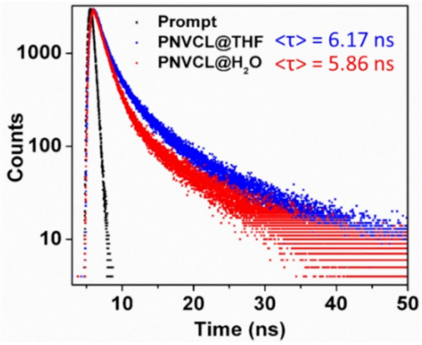

Fig. S5 Time-resolved luminescence decay of PNVCL in THF and water. The average lifetime was calculated using the biexponential decay equation: $\langle\tau\rangle = \dfrac{\alpha_1\tau_1^{2} + \alpha_2\tau_2^{2}}{\alpha_1\tau_1 + \alpha_2\tau_2}$

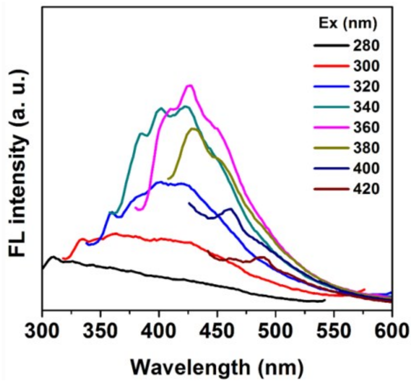

Fig. S6 Emission spectra of PNVCL monitored at different excitation wavelengths (λ_{ex}s) in water.

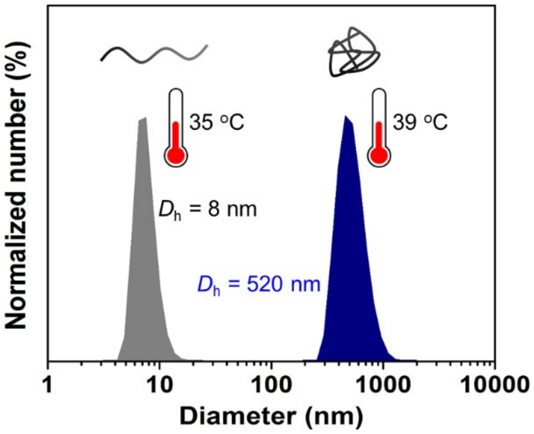

Fig. S7 Size distribution curves of 0.1 wt % aqueous solution of PNVCL at 35 and 39 °C.

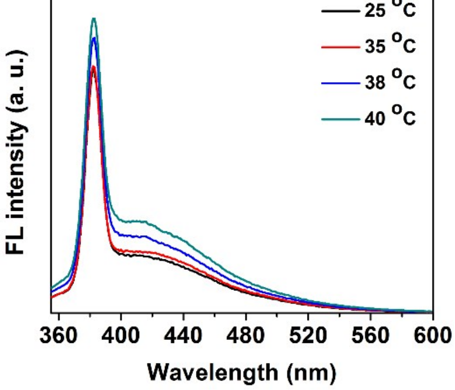

Fig. S8 Temperature-dependent fluorescence behaviour of 0.1 wt % PNVCL in water.

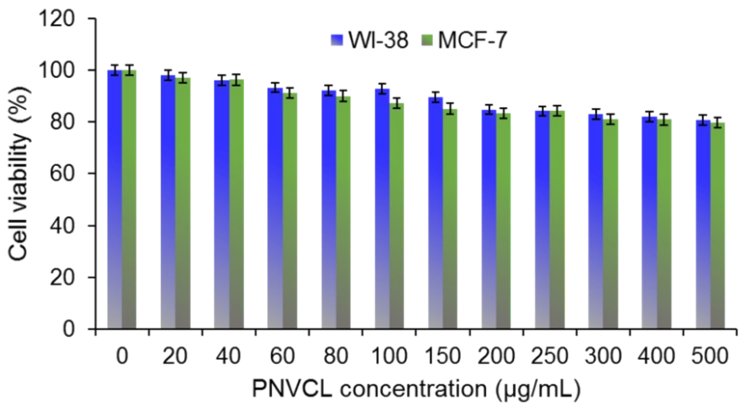

Fig. S9 *In vitro* cytotoxicity of PNVCL against WI-38 and MCF-7 cells with different concentrations at 37 °C.

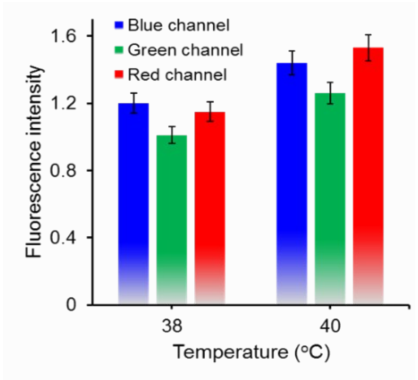

Fig. S10 Comparison of temperature induced fluorescence intensities of PNVCL treated MCF-7 cells at 38 and 40 °C for 24 h.

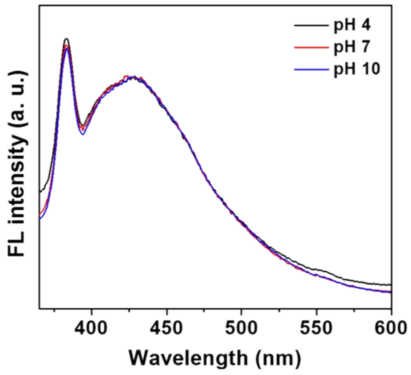

Fig. S11 Effect of pH on the fluorescence intensity of PNVCL. Concentration = 5 mg/mL.

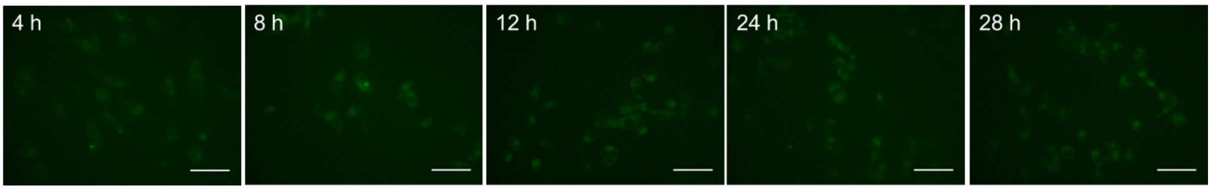

Fig. S12 Fluorescence microscopy images of MCF-7 cells treated with 250 μg/mL of PNVCL for different time intervals at 25 °C. Scale bar = 50 μm.

References

(1) D. B. G. Williams and M. Lawton, *J. Org. Chem.*, 2010, **75**, 8351-8354.

(2) S. Pal, S. G. Roy and P. De, *Polym. Chem.*, 2014, **5**, 1275-1284.

(3) S. S. Chaudhury, A. Sannigrahi, M. Nandi, V. K. Mishra, P. De, K. Chattopadhyay, S. Mishra, J. Sil and C. D. Mukhopadhyay, *Mol. Neurobiol.*, 2019, **56**, 6551-6565.

(4) B. Ruidas, S. S. Chaudhury, K. Pal, P. K. Sarkar and C. D. Mukhopadhyay, *Nanomedicine*, 2019, **14**, 1173-1189.
